# Supplementary material for: Attention deficit hyperactivity disorder and educational level in adolescent and adult individuals after anesthesia and abdominal surgery during infancy
Source: PLoS One. 2020 Oct 21;15(10):e0240891. doi: 10.1371/journal.pone.0240891 (PMC7577494; doi:10.1371/journal.pone.0240891)
Supplement: S1 File — (DOCX) [file pone.0240891.s001.docx]

| **Excluding codes for abdominal surgery** |
| --- |
| **Classification of Surgery years 1963-1996** |
| JAG10, JAG20, JAG60, JAG96, JAH00, JAH20, JAM10, JAW96, JBB10, JBB96, JBC00, JDB00, JDH00, JDH50, JDH60, DH61, JEA00, JEA10, JEW96, JFA00, JFA10, JFA17, JFA70, JFA73, JFA80, JFA83, JFA96, JFB00, JFB20, JFB30, JFB33, JFB40, JFB43, JFB46, JFB50, JFB60, JFB63, JFB96, JFC00, JFC10, JFC20, JFF13, JFF23, JFF26, JFF30, JFF96, JFK00, JFK01, JFK10, JFK96, JFK97, JFL00, JFL10, JFW96, JGC40, JJA23, JJA96, JJB40, JJB50, JJB96, JJW96, JKW96 |
| **Classification of Surgical Procedures from 1997** |
| 0058, 0059, 4010, 4011, 4045, 4270, 4272, 4290, 4440, 4410, 4429, 4499, 4510, 4511, 4599, 4600, 4611, 4681, 4740, 4741, 4630, 4631, 4640, 4641, 4642, 4643, 4644, 4649, 4621, 4730, 4739, 4459, 4660, 4661, 4668, 4700, 4710, 4711, 4713, 4721, 4770, 4771, 4130, 4781, 4782, 4821, 5180, 5112, 5119, 5339 |
