# Supplementary material for: Attention deficit hyperactivity disorder and educational level in adolescent and adult individuals after anesthesia and abdominal surgery during infancy
Source: PLoS One. 2020 Oct 21;15(10):e0240891. doi: 10.1371/journal.pone.0240891 (PMC7577494; doi:10.1371/journal.pone.0240891)
Supplement: S2 File — (DOCX) [file pone.0240891.s002.docx]

**Overview of the cohort**

The control group was drawn at random by statisticians at the Register service unit. When recieving the data from the registers the personal identification number was replaced by a serial number due to confidentiality. In the control group 15 individuals had the same serial number due to inaccurate personal identification numbers, therefore no data would be found on these individuals. 12 were matched to different cases and 3 were matched to the same case.

| **Number of controls per case** | **Number of cases** | **Number of controls in total** | **Number of total (cases+controls)** |
| --- | --- | --- | --- |
| 10 | 472 | 4 720 | 5 192 |
| 9 | 12 | 108 | 120 |
| 7 | 1 | 7 | 8 |
| **Total** | **485** | **4 835** | **5 320** |

**Highest level of education**

There are 31 cases and 399 controls that miss the outcome of highest educational level. With a case missing this outcome also its controls were excluded in the analysis.

| **Number of controls per case** | **Number of cases** | **Number of controls in total** | **Number of total (cases + controls)** |
| --- | --- | --- | --- |
| 10 | 264 | 2 640 | 2 904 |
| 9 | 125 | 1 125 | 1 250 |
| 8 | 39 | 312 | 351 |
| 7 | 10 | 70 | 80 |
| 6 | 4 | 24 | 28 |
| 5 | 6 | 30 | 36 |
| 4 | 4 | 16 | 20 |
| 3 | 1 | 3 | 4 |
| 2 | 0 | 0 | 0 |
| 1 | 1 | 1 | 2 |
| **Total** | **454** | **4 221** | **4 675** |

**Income**

There are 2 cases and 325 controls that miss the outcome of income. With a case missing this outcome also its controls were excluded in the analysis.

| **Number of controls per case** | **Number of cases** | **Number of controls in total** | **Number of total (cases + controls)** |
| --- | --- | --- | --- |
| 10 | 280 | 2 800 | 3 080 |
| 9 | 133 | 1 197 | 1 330 |
| 8 | 44 | 352 | 396 |
| 7 | 8 | 56 | 64 |
| 6 | 9 | 54 | 63 |
| 5 | 3 | 15 | 18 |
| 4 | 3 | 12 | 15 |
| 3 | 2 | 6 | 8 |
| 2 | 0 | 0 | 0 |
| 1 | 1 | 1 | 2 |
| **Total** | **483** | **4 493** | **4 976** |
