# Supplementary material for: Attention deficit hyperactivity disorder and educational level in adolescent and adult individuals after anesthesia and abdominal surgery during infancy
Source: PLoS One. 2020 Oct 21;15(10):e0240891. doi: 10.1371/journal.pone.0240891 (PMC7577494; doi:10.1371/journal.pone.0240891)
Supplement: Attachment — Submitted filename: Response to reviewers.docx [file pone.0240891.s003.docx]

**Regarding manuscript PONE-D-20-10556
Exposure to anesthesia and abdominal surgery during infancy is not associated with cognitive dysfunction later in life**

**PLOS ONE**

Dear Academic Editor.

Thank you for your valuable comments regarding the manuscript. We have taken careful consideration of all the comments and the manuscript has been changed according to yours and the reviewers suggestions. We think that the manuscript has improved and hope that it can be accepted for publication in PLOS ONE. Please see our response on the comments listed below.

Our manuscript meets PLOS ONE's style requirements, including those for file naming. We have rephrased and omitted some minor occurrence of overlapping text with a previous publication and we have made data underlying the findings in the manuscript fully available as supporting information.

Sincerely

Cecilia Arana Håkanson, corresponding author

Department of Women's and Children's Health, Uppsala University.

e-mail: cecilia.arana_hakanson@kbh.uu.se

**Response to reviewers**

**Reviewer #1:** *This is case control study to evaluate whether exposure to anesthesia and abdominal surgery during infancy was associated with attainment in educational level and income in adult lives. The strengths of the study is that a uniform type of surgery was performed. However, although the study adds information to this literature, the title of the paper is mis-leading as the study is mis-leading and should be revised to properly reflect what was done*.

**Response:** We have changed the title to “Attention deficit hyperactivity disorder and educational level in adolescent and adult individuals after abdominal surgery during infancy”

*There are a few areas that need further discussion:
1. The study was conducted over a long period of time. Whether changing practice (such as surgical approaches or anesthetic management) might have affected the outcomes was not discussed.*

**Response:** The study was conducted over a long period of time and both surgical approaches and anesthetic management have changed over that period. However, our study uses a case-control approach with the individuals being exposed to abdominal surgery during infancy and the controls not. The cases are not compared to each other but only to their controls matched on sex, age and gestational week. Therefore, these changes should not affect the outcome.

*2. Measurement of ADHD - it is not clear whether it was well captured, as likely not all patients with ADHD sought medical consultation.*

**Response:** The measurement of ADHD was drawn from the Swedish National Patient Register, hence we do miss individuals that does not seek medical consultation. However, we believe that there should not be a difference in the pattern of seeking medical consultation between the cases and their controls.

*3. Age of exposure – it appears that the majority of the subjects were exposed to anesthesia and surgery at a later gestational age (>33 weeks). Whether these results can be generalized to those exposure at much earlier gestational age need to be included in the discussion.*

**Response:** We have in the discussion page 16, lines 312-314 added “As the majority of neonates were exposed at a later gestational age (>33 weeks) the results regarding exposure at much earlier gestational age might be different and can not be generalized”.

*4. Similarly, the number of repeated exposures to anesthesia and surgery was also not high. It is unclear whether the authors only measured repeated abdominal surgery, and not other type of surgery.*

**Response:** Only exposure to abdominal surgery during infancy were included and no other type of surgery or other procedures requiring anaesthesia. This information is added in Study Cohort page 6 ,lines 122-123.

***Reviewer #2****: This is an interesting manuscript evaluating a number of different outcomes in children exposed to anesthesia for abdominal surgery.

Specific comments:
Page 4, Line 73: While some studies have found a near doubling of ADHD, this was only found in the children with multiple exposures. In the same study by Sprung et al., the HR for a single exposure was 1.18, which was not statistically significant. In more recent studies published in Anesth Analg. by Ing et al. the HRs for an increased risk of ADHD was found to be between 1.25 and 1.37.*

**Response:** Page 4, lines 76-78 we have added **repeated** to the sentence ”Previous studies have found a doubling of the incidence of learning disabilities (LD) and attention deficit hyperactivity disorders (ADHD) after **repeated** exposure to general anesthesia before age 2-4 years (1-3). We also added this sentence: ”In a more recent study, an increased risk of ADHD was observed in children under age 5 with a single exposure to minor surgery requiring anesthesia” (4).

*Page 7, Line 131: It would be helpful to know how many total patients were in the Swedish Medical Birth Register and how they were drawn at random based on sex, age, and gestational week. Were they manually selected by one of the researchers or was there a computer program that would randomly select from anywhere in the database?*

**Response:** The Swedish Medical Birth Register includes 98% of all births in Sweden since 1973. Register data is protected by strict confidentiality but can be made available for research after a special application following ethical approval as in our study. The data withdrawal and the random selection were made by statisticians employed at the Register service unit using computer programs.

*Page 7, Line 133: Is it possible that the controls had surgery after age 1 year old? Therefore, if anesthetics had an adverse effect in children between ages 1 and 3 and the controls included children exposed at those ages, is it possible that these results may be biased towards a null effect?*

**Response:** Yes, there is a possibility that the controls and their cases had surgery and/or anesthetics after the age of one year. However we set the exposure of the cases to abdominal surgery before the age of one. Since the 4835 controls were drawn at random we believe that the group should reflect the common exposure to surgery and/or anesthetics in the Swedish population in general.

*Page 7, Line 147: Is there a reason why the 1995 cutoff was chosen?*

**Response:** The diagnose of ADHD is mainly found in the outpatient registers and this register started in 2001. The diagnose of ADHD is hard to confirm in very young children. With the cutoff 1995 the individuals were 6 years or older and more likely to have the diagnose code for ADHD in the register.
 *Page 7, line 148: It would be helpful to explain why mixed models are needed for these regression analyses. This data does not seem to be hierarchical in nature.*

**Response:** In order to be able to take the matching into account a mixed ordinal regression was estimated. Taking specific care by treating a case and its matched controls as a cluster would assist in preventing that the results could be biased by for example unequal number of controls between cases due to missing data. We have added this information under statistics pages 7-8, lines 155-159. *Page 8, Line 157: If there were 485 cases and it is 1:10 matching, shouldn’t there be 4850 controls instead of 4835?*

**Response:** A very valid comment. The control group was drawn at random by statisticians at the Register service unit. When recieving the data from the registers the personal identification number was replaced by a serial number due to confidentiality. In the control group 15 individuals had the same serial number due to inaccurate personal identification numbers, therefore no data would be found on these individuals. 12 were matched to different cases and 3 were matched to the same case. Please see the table below.

| **Number of controls per case** | **Number of cases** | **Number of controls in total** | **Number of total (cases+controls)** |
| --- | --- | --- | --- |
| 10 | 472 | 4 720 | 5 192 |
| 9 | 12 | 108 | 120 |
| 7 | 1 | 7 | 8 |
| **Total** | **485** | **4 835** | **5 320** |

*Figure 1: This flow diagram would be more informative if it also included the selection of the controls.*

**Response:** We have changed the flow diagram to include selection of the controls. *Table 1: It may be more informative to show the demographic characteristics of the 485 patients included in the study. I believe the 523 patients includes patients who were excluded due to chromosomal aberrations. If there was missing gestational age data in 10 patients, how was the matching done? Was matching done on only age and sex for those patients? In the children with multiple procedures (2 or 3 or more) were these all procedures done before age 1, or at any time during their life? It would also be helpful to note the demographic characteristics in the matched controls, which should confirm exact matching and give information on whether they had surgery or anesthesia later on in their life.*

Control cohort n=4850

Included in the control n=4835

12 cases with only 9 controls n=4838

1 case with only 7 controls n=4835

**Response:** Correct, the 523 patients include the excluded patients. We agree that it would be more informative with the demographic data on only the included 485 individuals. Due to confidentiality in the registers we do not have the information on which ones that were excluded and therefore we were not able to only show the demographic characteristics of only the 485 individuals. The missing gestational age in 10 individuals were when we manually collected the data from the local patients charts at the hospital. The matching of controls was made by the information in the Swedish Birth Register connected to the personal identification code of 10 digits that all citizens are given at birth and not from the data that were manually collected. All controls were matched on age, sex and gestational age (week). This study was based on a cohort from our previous study of 898 patients that had undergone laparotomy during the first year of life between the years 1976 tom 2011. 523 patients matched the criteria of being aged over 16 years at study start. The number of surgeries were the total number of surgeries during the follow up period of the previous study with a median follow up of 14.7 years (0.0 – 36.0). We did not collect register data on whether the controls were exposed to surgery and/or anesthesia later in life. In the children with multiple procedures (2 or 3 or more) only the first procedure had to be done before age 1. Most reoperations were done close in time from the primary surgery e.g 70% undergoing laparotomy for small bowel obstruction occurred within 2 years of the primary surgery.

*Table 2: It may be more informative to show the surgical characteristics of the 485 patients included in the study.*

**Response:** We agree that it would be more informative with the surgical characteristics on only the included 485 individuals. Due to confidentiality in the registers we do not have the information on which ones that were excluded and therefore we were not able to only show the data of the 485 individuals.

*Table 3: If there are n=485 exposed children and n=4835 matched controls, why is there only data for n=454 exposed children and n=4221 controls? Is there missing outcome data for the cases and controls?*

**Response:**

There are 31 cases and 399 controls that miss the outcome of highest educational level. With a case missing this outcome also its controls were excluded in the analysis. Please see the table below for further explanation.

| **Number of controls per case** | **Number of cases** | **Number of controls in total** | **Number of total (cases + controls)** |
| --- | --- | --- | --- |
| 10 | 264 | 2 640 | 2 904 |
| 9 | 125 | 1 125 | 1 250 |
| 8 | 39 | 312 | 351 |
| 7 | 10 | 70 | 80 |
| 6 | 4 | 24 | 28 |
| 5 | 6 | 30 | 36 |
| 4 | 4 | 16 | 20 |
| 3 | 1 | 3 | 4 |
| 2 | 0 | 0 | 0 |
| 1 | 1 | 1 | 2 |
| **Totalt** | **454** | **4 221** | **4 675** |

*Table 4: It would be helpful to define IRR.*

**Response:** IRR, incidence rate ratio shows the probability of having a higher level of education. Added in the statistics section page 7,lines 153-154.

*Table 7: If there are n=485 exposed children and n=4835 matched controls, why is there only data for n=431 exposed children yet there is data for all n=4835 controls? If this is missing diagnosis data, is there a reason why it is only missing in the exposed children and not the matching controls?*

**Response:** Thank you for your careful review, we made a mistake here. The right number of cases is 460 (and not 406) with no ADHD diagnosis and 25 with the diagnosis. We must have mixed up the numbers when editing the table. Now corrected in the Table 7.

*Page 14, Line 279: Given that the incidence of ADHD diagnosis has increased over time, it would be interesting to know if the birth years of the exposed and matched controls were similar. If they were born in different eras, it may bias the results.*

**Response:** The matching of age was made by the same year of birth for cases and controls so this would not be a source of bias.

*Page 15, Line 298: While the exposed children and matched controls were matched on age, sex, and gestational week, is it possible that there may be other important differences between the groups that were not measured such as socioeconomic status of the families or baseline health characteristics?*

**Response:** We are aware that there may be other important differences between the groups that were not measured such as socioeconomic status of the families or baseline health characteristics. The matching could have been made with more potential confounders but it would decrease the number of individuals in the control group. Matching by gestational age had higher priority in our study. We have added this section in discussion page 14, lines 272-276.

1.	Sprung J, Flick RP, Katusic SK, Colligan RC, Barbaresi WJ, Bojanic K, et al. Attention-deficit/hyperactivity disorder after early exposure to procedures requiring general anesthesia. Mayo Clin Proc. 2012;87(2):120-9.

2.	Flick RP, Katusic SK, Colligan RC, Wilder RT, Voigt RG, Olson MD, et al. Cognitive and behavioral outcomes after early exposure to anesthesia and surgery. Pediatrics. 2011;128(5):e1053-61.

3.	Wilder RT, Flick RP, Sprung J, Katusic SK, Barbaresi WJ, Mickelson C, et al. Early exposure to anesthesia and learning disabilities in a population-based birth cohort. Anesthesiology. 2009;110(4):796-804.

4.	Ing C, Sun M, Olfson M, DiMaggio CJ, Sun LS, Wall MM, et al. Age at Exposure to Surgery and Anesthesia in Children and Association With Mental Disorder Diagnosis. Anesth Analg. 2017;125(6):1988-98.
