# Supplementary material for: Attention deficit hyperactivity disorder and educational level in adolescent and adult individuals after anesthesia and abdominal surgery during infancy
Source: PLoS One. 2020 Oct 21;15(10):e0240891. doi: 10.1371/journal.pone.0240891 (PMC7577494; doi:10.1371/journal.pone.0240891)
Supplement: Attachment — Submitted filename: Response to reviewers.docx [file pone.0240891.s004.docx]

**Regarding PONE-D-20-10556R1
Attention deficit hyperactivity disorder and educational level in adolescent and adult individuals after anesthesia and abdominal surgery during infancy
PLOS ONE**

Dear Academic Editor

Thank you for the valuable comments regarding the manuscript. We have taken careful consideration to all the comments and the manuscript has been changed according to the reviewers’ suggestions. We believe that the manuscript has improved further and hope that it can be accepted for publication in PLOS ONE.

Sincerely

Cecilia Arana Håkanson, corresponding author

Department of Women's and Children's Health, Uppsala University.

e-mail: cecilia.arana_hakanson@kbh.uu.se

**Review Comments to the Author**

*Reviewer #2: The authors did a nice job answering the queries and comment. There are however a few additional comments and follow-up concerns based on the author’s responses.*

*Page 8, line 162: If there is inaccuracy in the ID numbers received from the Register service and those controls had to be excluded, that is a valid reason why there are fewer than 10 controls for each case. This also justifies why it would be important to analyze these groups in clusters since there are missing patients from some of the matched sets. It is unlikely that this would markedly influence the results, but this should however be disclosed and explained to the reader in the Results and Discussion.*

**Response**: We have added this extra information plus an explaining table (Table 1) in Results page 8 lines 164-166 and in the discussion section page 16 lines 319-321.

*Figure 1b: If possible, it would be preferable to combine both cases and controls into one flow diagram in order for the reader to appreciate where the cases and matched controls originated from. From the new diagram it appears that the exclusion criteria of no chromosomal aberrations was applied to the cases, but not to the control patients. However in the text (Page 7, line 143) it seems that this exclusion criteria was made. It would be helpful to include this in the flow diagram so the readers can see that the same exclusions were made to both cohorts.*

**Response**: Thank you for this input. We have now made a new flow diagram which replaces Fig 1a and 1b.

*Table 1: Thank you for the clarification. It makes sense that if the authors cannot differentiate the patients, it would be impossible to give the patients characteristics for only the985 included individuals. However, this should be disclosed in a footnote for this demographic table, that the table includes data for 38 patients who were ultimately excluded from analysis due to chromosomal anomalies. Otherwise it would be confusing to the reader why the numbers do not match up.*

**Response:** The information that it includes individuals that later were excluded for chromosomal aberration is already in the table. However, we now added the number, 38, to the table footnote to make it clearer to the reader plus added an extra asterix to “highlight the information”.

*Page 9, line 172 and Table 1: It should also be mentioned that the additional surgeries did not necessarily occur during the study period, and could have occurred at any time during the follow-up period which was between 0 and 36 years of age. Otherwise it looks like your exposure cohort had a multiple surgery rate of nearly 50% at under 1 year of age.*

**Response:** We have added this information to the table as a footnote as well as on page 9 line 179.

*Table 2: It should also be disclosed that this table of procedures and operating room times included procedures for 38 patients who were excluded from analysis as a footnote in this table.*

**Response:** We have added this information to the table as a footnote.

*Page 7, line 140: The authors mention that they matched on age, sex, and gestational week. In their response to a reviewer comment, they mention that they also match on year of birth to ensure that children were born in the same era. If year of birth is an additional matching criteria, it would be helpful to mention this in the text. Also if the authors have data on these variables for the cases and controls including the year of birth, it may be helpful to add this data to the Table 1 patient characteristics table.*

*Ideally it would be helpful to display the characteristics for the controls in a patient characteristics table. Is that possible, or do the authors not have any data for the controls other than ID numbers to match to their cases? Also It seems that authors cannot identify which cases got matched since they were unable to display the characteristics of which of the 523 cases were chosen and which were excluded. I believe the Swedish Birth Register data has been widely used and is likely to be reliable. However, these aspects of the study should likely be mentioned if this is the case.*

**Response:** The year of birth is not an additional matching criteria, it is the same criteria as the criteria for matching on age. We have also added information on median age and median gestational week of the cases and control group to page 8 lines 167-169

*Page 7, line 155: If a mixed ordinal regression was used to take into account matched sets and missing controls in the primary analysis, shouldn’t a similar analysis be used in the ADHD analysis instead of Fisher’s exact tests since the title of the study is now the evaluation of ADHD?*

**Response:** In theory it would be possible to analyze ADHD using a mixed logistic regression, however limitations in the data causes issues with convergence when estimating the model. The issue is caused due to the few number of ADHD diagnoses in the group of cases.
